# Supplementary material for: Resolving the full spectrum of human genome variation using Linked-Reads
Source: Genome Res. 2019 Apr;29(4):635–45. doi: 10.1101/gr.234443.118 (PMC6442396; doi:10.1101/gr.234443.118)
Supplement: Supplemental Material [file supp_gr.234443.118_Supplemental_longranger-2.2.zip › longranger-2.2.tar.bz2!/lr2.2-public/lib/go/src/code.google.com/vitess/third_party/py/mock-1.0.1.tar.gz!/mock-1.0.1/html/genindex.html]

Index — Mock 1.0.1 documentation


### Navigation

- index
- Mock 1.0.1 documentation »

# Index

**\_**
| **A**
| **C**
| **D**
| **E**
| **F**
| **G**
| **H**
| **I**
| **M**
| **N**
| **O**
| **P**
| **R**
| **S**
| **T**
| **U**
| **W**

## \_

|  |  |
| --- | --- |
| \_\_call\_\_  \_\_class\_\_ (Mock attribute) | \_\_dir\_\_() (Mock method)  \_get\_child\_mock() (Mock method) |

## A

|  |  |
| --- | --- |
| ANY (in module mock)  articles  assert\_any\_call() (Mock method)  assert\_called\_once\_with() (Mock method) | assert\_called\_with() (Mock method)  assert\_has\_calls() (Mock method)  attach\_mock() (Mock method) |

## C

|  |  |
| --- | --- |
| call() (in module mock)  call\_args (Mock attribute)  call\_args\_list (Mock attribute)  call\_count (Mock attribute)  call\_list() (call method) | called (Mock attribute)  calling  configure\_mock() (Mock method)  create\_autospec() (in module mock) |

## D

DEFAULT (in module mock)

## E

easy\_install

## F

FILTER\_DIR (in module mock)

## G

Getting Started

## H

hg

## I

|  |  |
| --- | --- |
| installing | introduction |

## M

|  |  |
| --- | --- |
| MagicMock (class in mock)  method\_calls (Mock attribute)  Mock (class in mock)  mock (module) | mock\_add\_spec() (Mock method)  mock\_calls (Mock attribute)  mock\_open() (in module mock) |

## N

|  |  |
| --- | --- |
| name  NonCallableMagicMock (class in mock) | NonCallableMock (class in mock) |

## O

older versions

## P

|  |  |
| --- | --- |
| patch() (in module mock)  patch.dict() (in module mock)  patch.multiple() (in module mock)  patch.object() (in module mock) | patch.stopall() (in module mock)  pip  PropertyMock (class in mock)  Python 3 |

## R

|  |  |
| --- | --- |
| references  repository | reset\_mock() (Mock method)  return\_value  (Mock attribute) |

## S

|  |  |
| --- | --- |
| sentinel (in module mock)  setuptools | side\_effect  (Mock attribute)  spec |

## T

tests

## U

unittest2

## W

wraps

### Quick search


Enter search terms or a module, class or function name.

### Navigation

- index
- Mock 1.0.1 documentation »

© Copyright 2007-2012, Michael Foord & the mock team.
Last updated on Nov 05, 2012.
Created using Sphinx 1.1.3.
